# Regret and Therapeutic Decisions in Multiple Sclerosis Care: Literature Review and Research Protocol

**DOI:** 10.3389/fneur.2021.675520

**Published:** 2021-06-21

**Authors:** Gustavo Saposnik, Guillermo Bueno-Gil, Ángel P. Sempere, Alfredo Rodríguez-Antigüedad, Beatriz del Río, Mar Baz, María Terzaghi, Javier Ballesteros, Jorge Maurino

**Affiliations:** ^1^Laboratory for Social and Neural Systems Research, Department of Economics, University of Zurich, Zurich, Switzerland; ^2^Clinical Outcomes & Decision Neuroscience Unit, Li Ka Shing Institute, University of Toronto, Toronto, ON, Canada; ^3^Division of Neurology, Department of Medicine, St. Michael's Hospital, University of Toronto, Toronto, ON, Canada; ^4^Medical Department, Roche Farma, Madrid, Spain; ^5^Department of Neurology, Hospital General Universitario de Alicante, Alicante, Spain; ^6^Department of Neurology, Hospital Universitario Cruces, Bilbao, Spain; ^7^Department of Neurology, Hospital Universitario de La Princesa, Madrid, Spain; ^8^Department of Psychiatry, Hospital Universitari Vall d'Hebrón, Barcelona, Spain; ^9^Department of Neurosciences and CIBERSAM, University of the Basque Country (UPV/EHU), Leioa, Spain

**Keywords:** multiple sclerosis, regret, decision making, healthcare professionals, neurologists, nurses

## Abstract

**Background:** Decisions based on erroneous assessments may result in unrealistic patient and family expectations, suboptimal advice, incorrect treatment, or costly medical errors. Regret is a common emotion in daily life that involves counterfactual thinking when considering alternative choices. Limited information is available on care-related regret affecting healthcare professionals managing patients with multiple sclerosis (MS).

**Methods:** We reviewed identified gaps in the literature by searching for the combination of the following keywords in Pubmed: “regret and decision,” “regret and physicians,” and “regret and nurses.” An expert panel of neurologists, a nurse, a psychiatrist, a pharmacist, and a psychometrics specialist participated in the study design. Care-related regret will be assessed by a behavioral battery including the standardized questionnaire Regret Intensity Scale (RIS-10) and 15 new specific items. Six items will evaluate regret in the most common social domains affecting individuals (financial, driving, sports—recreation, work, own health, and confidence in people). Another nine items will explore past and recent regret experiences in common situations experienced by healthcare professionals caring for patients with MS. We will also assess concomitant behavioral characteristics of healthcare professionals that could be associated with regret: coping strategies, life satisfaction, mood, positive social behaviors, occupational burnout, and tolerance to uncertainty.

**Planned Outcomes:** This is the first comprehensive and standardized protocol to assess care-related regret and associated behavioral factors among healthcare professionals managing MS. These results will allow to understand and ameliorate regret in healthcare professionals.

**Spanish National Register** (SL42129-20/598-E).

## Introduction

Decision making in medical care is a complex, cognitively and emotionally demanding task (1). The current therapeutic landscape for patients with chronic conditions is evolving as a result of the challenges imposed by population aging and prolonged life expectancy leading to a higher prevalence of patients with multiple comorbidities. Advances in therapeutic options with different safety and efficacy profiles and higher clinical demands add other challenges to healthcare professionals when facing diagnostic or therapeutic choices. Decisions influenced by cognitive biases or emotions may result in unrealistic patient and family expectations, incorrect advice, or suboptimal treatment decisions, leading to poorer clinical outcomes (2, 3).

Regret is a cognitive emotion that involves counterfactual thinking, when considering alternative choices (4). In other words, regret is an emotion experienced when one believes that the current situation would have had a better outcome by choosing a different course of action (e.g., alternative treatment or intervention) (5). Healthcare professionals are vulnerable to regret given their limited education in both decision making and risk management at medical schools (2).

Despite significant advances in patient care, the role of emotions in therapeutic decisions has not been extensively investigated. Studying regret is interesting because it involves the cognitive and affective component of medical decisions. Moreover, studies using functional magnetic resonance imaging have identified specific pathways, including activation of the medial orbitofrontal cortex, left superior frontal cortex, right angular gyrus, and left thalamus, which correlates with the degree of regret (5, 6). It is precisely those brain regions that are involved in decision making under uncertainty.

The consequences of regret in medicine include negative health outcomes among healthcare professionals, such as a high number of days with back pain and sleep problems, poor health-related quality of life, high sick leave days, and low job satisfaction with high turnover (7–12).

The aim of this article is to inform physicians and researchers about a standardized protocol using validated scales to assess regret associated with clinical decisions and to conduct a review of the medical literature. We focused on multiple sclerosis (MS) for being the paradigm of neurological conditions with an uncertain disease trajectory and a broad spectrum of therapeutic options, which carry consequences for patients and their families (e.g., disability at young age, cognitive impairment, and impaired quality of life) (13). Treatment selection (early high-efficacy therapies vs. escalation from a low to a higher efficacy treatment) represents the current challenge faced by clinicians (14, 15). This scenario is a common ground for the appearance of emotional regret at both extremes of treatment modalities (e.g., side effects due to early selection of a high-efficacy treatment or progression of disability when a treatment escalation approach is selected). To the best of our knowledge, no study has yet investigated care-related regret in the context of MS management.

## Methods

### Study Design

The DECISIONS-MS is a non-interventional, prospective, web-based study to assess emotional regret associated with treatment decisions in MS care. We first identified gaps in the literature by conducting a systematic review (see section below) to design a comprehensive protocol to assess care-related regret by healthcare professionals managing patients with MS. This study will be conducted in accordance with the Good Clinical Practice Guidelines of the International Conference on Harmonization and with the ethical principles of the Declaration of Helsinki and was approved by the institutional review board of the Hospital Clínico San Carlos (Madrid, Spain; reference number: 20/598-E). Informed consent will be obtained from all participants.

### Participants

Practicing healthcare professionals actively involved in the care of patients with MS in Spain will be invited to participate in our study by the Spanish Societies of Neurology and Neurology Nurses (SEN and SEDENE, respectively). Exclusion criteria will be healthcare professionals not currently involved in patient care or retired. Participants will receive an honorarium at the end of the study completion in recognition for the time and effort they provided to collaborate in this study.

### Outcome Measures

**Figure 1:**
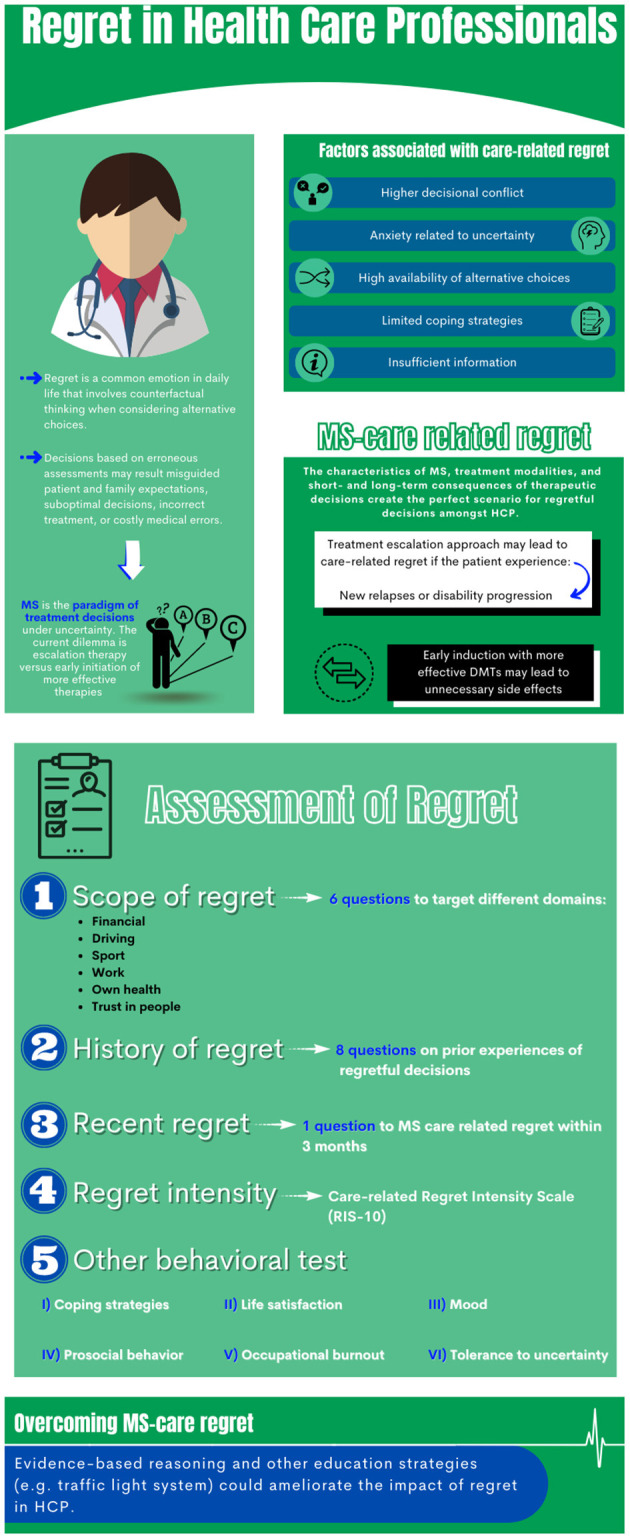
Regret in medicine.

Care-related regret will be assessed through the combination of the standardized questionnaire Regret Intensity Scale (RIS-10) and a battery of 15 specific items designed by a research team of neurologists, a nurse, a psychiatrist, a pharmacist, and a psychometrics specialist (Figure 1). We applied a comprehensive framework to study regret, acknowledging the most common social domains affecting individuals: financial, driving, sports/recreation, work, own health, and confidence in people. This strategy was derived from the Socio-Economic Panel (SOEP), a longitudinal survey to study risk and behavioral preferences in a population (16).

The RIS-10 is a self-reported, generic questionnaire to measure the affective, physical, and cognitive intensity of regret among healthcare professionals (17). Items are assessed with a five-point Likert scale ranging from 1 (“not at all”) to 5 (“absolutely”). Higher scores indicate greater intensity of regret. The RIS-10 possesses good psychometric properties, with an internal consistency of 0.87 (Cronbach's alpha) and a test–retest reliability of 0.70 (17).

The 15-item collection designed by our team aims to assess regret behaviors in a broad spectrum of hypothetic scenarios, including daily activities and MS patient care-related tasks. The first six questions assess the intensity of regret in daily episodes along different domains: financial, driving, sports–recreation, work, own health, and confidence in people. Respondents must score on a scale from 0 (little affected) to 10 (very affected), the degree of regret experienced in the situation evoked by each item. If participants report no regret, a mean intensity of 0 will be imputed. The next eight items assess whether the participant has faced common situations experienced by healthcare professionals managing patients with MS in clinical practice. A final item assesses the presence of regret related to MS care in the last 3 months. Intensity of regret is also reported in items 8–15: the participant must rank on a scale from 0 (little affected) to 10 (very affected) the degree of regret if the situation has been encountered.

Given that factors associated with regret include behavioral characteristics of healthcare providers, we also propose assessing six relevant domains: life satisfaction, mood, coping strategies, prosocial behavior, occupational burnout, and reaction to uncertainty (18–23).

The Satisfaction with Life Scale (SWLS) is a five-item scale designed to measure global cognitive judgments of one's life satisfaction (18). Participants indicate how much they agree or disagree with each item using a seven-point scale that ranges from strongly agree to strongly disagree. Scores between five to nine indicate that the respondent is extremely dissatisfied with life, whereas scores between 31 to 35 indicate that the respondent is extremely satisfied.

The Beck Depression Inventory—Fast Screen (BDI-FS) is a self-report, seven-item questionnaire assessing the level of depressive symptoms (19). Responses to the items are provided on a four-point scale (no symptoms to severe symptoms). Total scores range from 0 to 21, with higher scores indicating greater severity of depressive symptoms. Cut-off scores ≥4 and ≥9 are used to define the presence of depression and moderate-to-severe depression, respectively.

Coping strategies will be assessed using the Brief-COPE (Coping Orientation to Problems Experienced Inventory) (20). Participants are asked to recall the most serious stressor they have experienced during the previous year and to indicate how they responded to it. This questionnaire consists of 28 items that measure 14 factors of two items each on a Likert scale ranging from 1 = “I didn't do this at all” to 4 = “I did do this a lot.” Venting, denial, substance use, behavioral disengagement, self-distraction, and self-blame are considered maladaptive coping strategies, while positive reframing, planning, seeking emotional support, active coping, instrumental support, acceptance, religion, and humor are adaptive strategies. Maladaptive strategies are more closely related with mental health problems such as perceived stress and depression (24). Conversely, adaptive strategies are more related with psychological well-being and life satisfaction.

The Prosocial Behavioral Intentions Scale (PBIS) will be used to measure positive social behaviors (21). It is a four-item unidimensional measure with a seven-point Likert-type scale (1 = “Definitely would not do this” to 7 = “Definitely would do this”). No items are reverse scored. The possible scores range from 4 to 28, with higher scores indicating higher levels of prosocial behavioral intentions.

Occupational burnout will be assessed using the Maslach Burnout Inventory—Human Services Survey for Medical Personnel (MBI-HSS) (22). This instrument measures burnout as a continuum, ranging from low to high, on three different dimensions: emotional exhaustion (nine items), depersonalization (five items), and personal accomplishment (eight items). Each item scores from 0 = “never” to 6 = “every day.”

Finally, we used the “tolerance to uncertainty” in patient care, using the physician's reaction to uncertainty test (23). A shorter version following a factor analysis comprises five questions showing reliable psychometric properties (α-Cronbach 0.90). Participants rate the level of agreement with the following statements from 0 (strongly disagree) to 5 (strongly agree): (i) the uncertainty of patient care often troubles me; (ii) I find the uncertainty involved in patient care disconcerting; (iii) I usually feel anxious when I am not sure of the diagnosis; (iv) uncertainty in patient care makes me uneasy; and (v) I am quite comfortable with the uncertainty in patient care. Note that the last item is reverse coded for consistency. After participants provide a rating for each question, all are added to obtain a total score (25). Previous studies have shown that physician's low tolerance to uncertainty was associated with higher resource utilization and patients being recalled for studies, as well as treatment inertia (26–29).

Socio-demographic and work-related characteristics at baseline will be collected to identify the potential association with care-related regret (Table 1).

**Table 1:** Socio-demographic characteristics, professional background, and practice setting.

•Age •Gender •Years of experience as a healthcare professional •Neurologist or nurse •Specialization in MS •Years of experience managing MS •Practice setting (academic or non-academic hospital) •Number of MS patients seen per week •Co-investigator in clinical trials •Co-author in a peer-reviewed publication within the last year

*MS, multiple sclerosis*.

Figure 1 shows a summary of the protocol and behavioral battery.

### Follow-Up

The same behavioral battery will be assessed 6 months, 1 year, and 2 years later to compare variability of patient-related regret and its impact on therapeutic decisions.

### Statistical Analysis

Data analyses will be performed using Stata 16® statistical software. All patients participating in the study who meet the eligibility criteria will be included in the study population. Continuous data will be presented as number of observations (N), mean, standard deviation (SD), minimum, Q1, median, Q3, and maximum. Data for categorical and ordinal variables will be presented as counts, proportions, or percentages. In both cases, the number of missing data (N missing) will be specified.

Factors associated with care-related regret will be assessed in multivariate analysis adjusting for demographic factors and behavioral characteristics of participants (e.g., coping, occupational burnout, life satisfaction, tolerance to uncertainty, depression, and prosocial behavior). Similarly, we will investigate the role of care-related regret in treatment decisions (e.g., treatment inertia, suboptimal decisions) as described in our previous publications (28, 29). All statistical tests will be two-sided with an α-level of 0.05.

### Psychometric Analysis

The dimensional structure and item characteristics of the RIS-10 in the management of MS will be explored. A non-parametric item response theory (IRT) procedure, the Mokken scale analysis, will be performed to assess the underlying dimensions of the RIS-10 (30). Each of the 10 items will be required to have a scalability coefficient (Hi) of ≥0.30 and an overall scale scalability index (H) of ≥0.30 (31). A parametric Samejima's Grade Response Model will be conducted to further assess the information and discrimination of RIS-10 items. The variability of the RIS-10 scores will be assessed across the battery of new items created in this study to check for invariance or sensible ordering. All analyses will be performed with R v4.0.3 (https://cran.r-project.org/) using the mokken and ltm libraries.

### Sample Size

Our sample size calculation was based on the estimated prevalence of patient-care regret. Sorum et al. evaluated regret in American and French physicians ordering diagnostic test for the detection of prostate cancer (32). They identified that 60–70% of participants experience patient-care regret when facing 12 case scenarios. Another study found a similar prevalence of regret in Switzerland (33). We have eight variables that could be associated with care-related regret (e.g., age, sex, years of practice, coping, burnout, life satisfaction, depressive symptoms, and tolerance to uncertainty). Current guidelines on sample size for predictive models require a minimum of five events (i.e., regret and therapeutic inertia in our study) per predictor, yielding a minimum of 40 required events (34). We expect a 15% loss to follow-up. Thus, we need a minimum of 77 participants [40 events divided by 0.6 (incidence of regret) times 1.15 (to compensate for 15% loss to follow-up)]. An additional 77 participants will be recruited to allow for a potential lower incidence of regret and the potential inclusion of other covariates. Therefore, we plan to recruit a total of 154 participants.

### Systematic Literature Review

We completed a systematic review in order to identify gaps in the literature and improve the quality of our protocol. We expanded the previous systematic review (1979 to 2014) from Becerra Pérez et al. (35). We performed a search in PubMed using the following keyword combinations: “regret and decision,” “regret and physicians,” and “regret and nurses” comprising studies published from December 2014 until March 2021.

We included all studies that focused on care-related regret involving healthcare professionals (physicians and/or nurses) when making either hypothetical or “real life” healthcare decisions. Only original research articles were eligible; editorials, letters, abstracts, protocols, and systematic reviews were excluded.

### Results From the Systematic Review

The initial search using the aforementioned mesh terms yielded 652 articles. After the removal of duplicate publications and those not meeting our inclusion criteria, 14 articles remained for the analysis (Table 2) (8, 9, 11, 12, 33, 36–44). All articles included were published in English. Half of the studies (*n* = 7) were conducted in Switzerland (*n* = 4) or in the USA (*n* = 3). The mean age of participants ranged from 29 to 52 years. Of the 14 studies, four (28.6%) studies were exclusively conducted in physicians and only one study focused on nurses' regret; the remaining nine studies included regret in both healthcare professionals. Further details are summarized in Table 2.

**Table 2:** Literature review.

**Study**	**Country**	**Design**	**n**	**Mean age**	**Participants**			**Regret about…**	**Clinical context**	**Data collection**	**Response rate**	**Regret intensity assessment**	**Regret mean/median (SD. range)**	**alpha cronbach**	**Key findings**
					**Physician**	**Nurses**	**Others**								
Djulbegovic et al. (36)	US	Cross-sectional	221	31	100%	–	–	Care decisions	Residents, fellows and attending physicians	NA	NA	1 question (ranging 1–6)	2.45 (0.99)	NA	Negative correlation between regret and tendency for analytical thinking. Positive correlation between regret and maximizing, negative correlation with satisficing. Objectivism is a negative predictor of regret
Schmidt et al. (8)	Switzerland	Cross-sectional	460	39.5	47.8%	52.2%	–	Most important care-related regret event in the last 5 years	HCP from different clinical specialty	2011	31.2%	RIS-10	Physician = 1.70 (0.73) Nurses = 1.74 (0.65)	>0.85	Regret was associated with higher self-rated insomnia severity and sleeping pill use
Ben-Ezra and Bibi (37)	Israel	Cross-sectional	178	46.28	24.2%	36%	39.8%	Care-decisions during an armed conflict	HCP from different clinical specialty	July 2014	16.8%	DRS	8.75 (3.6)	0.878	Decision regret was positively associated with psychological distress and negatively associated with age
Cullati et al. (9)	Switzerland	Cross-sectional	775	39.5	39.9%	60.1%	–	Most important care-related regret event in the last 5 years	HCP from different clinical specialty	2011–2014	22.5%	RIS-10	Physician = 1.81 (0.76) Nurses = 1.86 (0.72)	0.87	Intensity of the most important regret in the previous 5 years was associated with poor SRH among both nurses and physicians, and with higher sick leave among nurses. Physicians may be better positioned than nurses to effectively cope with negative events
Richner et al. (33)	Switzerland	Cross-sectional	494	39.1	21.9%	78.1%	–	Most important care-related regret event in the last 5 years	HCP from different clinical specialty	NA	23.1%	RIS-10	2.04 (0.78)	German: 0.88 French: 0.87	The German version of the RIS is a valid and reliable instrument to assess regret intensity among HCP
Cheval et al. (10)	Multicentric (cohort form ICARUS study)	LongitudinalProspective	151	30.5	27.2%	48.3%	24.5%	Patient-care situations in the last week	Newly practicing HCP	2014–2017	NA	1 question (ranging 1-10)	Physician = 1.90 (2.15) Nurses = 1.69 (2.45) Others = 1.30 (2.03)	NA	Regret intensity has an immediate and a 1-week lagged influence on insomnia severity; regret accumulation had a lagged influence only. The associations between regret and insomnia severity is bidirectional
von Arx et al. (38)	Switzerland	Qualitative	24	37.2	45.8%	54.2%	–	Most important care-related regret event in the whole career	HCP from different clinical specialty	2016	50%	1 question (ranging 1–10)	7.3 (3.5–10)	NA	Most participants could easily identify one major healthcare-related regret in their work life. These regrets were often accompanied by serious emotional reactions and psychosomatic manifestations affecting their professional and private lives
Radhakrishnan et al. (39)	US	Cross-sectional	871	52.9	100%	–	–	Misdiagnosis of breast cancer	Internal Medicine, FM/GP and Gynecology	2016	52.3%	9 statements (5-point Likert scale)	Patients aged 45–49: 4.2 (0.8) Patients aged >75: 3.5 (0.9)	NA	Physicians were more motivated by potential regret in recommending screening for younger and older women than by concerns for patient-related hazards in screening. Regret varied according to physician specialty and guidelines most trusted
LeBlanc et al. (40)	US	Mixed methods	41	47.4	24.4%	–	75.6%	Treatment decision	Oncologists, patients and caregivers	2017	83.3%	DRS	15 (16.7)	NA	There were no statistical differences in regret, satisfaction, or conflict between groups
Cheval et al. (11)	Multicentric (cohort form ICARUS study)	Longitudinal Prospective	229	30	27%	48%	25%	Patient-care situations in the last week	Newly practicing HCP	2017–2018	NA	1 question (ranging 1–10)	Physician = 4.20 (1.41) Nurses = 4.18 (2.13) Others = 3.92 (1.79)	NA	Higher number or regrets was associated with job dissatisfaction, whereas more intense regrets were associated with increased turnover intention
Ibrahim et al. (12)	Multicentric (cohort form ICARUS study)	Longitudinal Prospective	105	29.7	–	100%	–	Patient-care situations in the last week	Newly practicing nurses	2017–2018	NA	1 question (ranging 1–10)	4.57 (2.29)	NA	Higher regret intensity was associated with an increased number of days with back pain in the following month. Negative association between number of regret experiences and decision to seek medical care
Boyle et al. (41)	Australia	Qualitative	399	NA	100%	–	–	Death after surgery	Surgeons	2007–2017	NA	Qualitative	In 16.9% of the cases, they would have acted differently	NA	Surgical decision-making may be accompanied by uncertainty that can lead to feelings of regret. Regret may be a relatively common response to adverse surgical events
Müller et al. (42)	Germany	Qualitative	29	NA	100%	–	–	Wrong diagnosis	Primary care physicians	2016	NA	Qualitative	Regret was present in 27 out of 29 cases	NA	Participants articulated regret, including strong emotions such as guilt and shame, irrespective of whether a different clinical course could have prevented harm
Cheval et al. (43)	Multicentric (cohort form ICARUS study)	LongitudinalProspective	276	30.4	29.0%	52.9%	18.1%	Patient-care situations in the last week	Newly practicing HCP	NA	NA	1 question (ranging 1–10)	Physician = 4.30 (2.05) Nurses = 4.03 (2.32) Others = 3.48 (1.67)	NA	Number (for nurses) and intensity (for physicians) of regrets were associated with an increased number of sick leaves

*DRS, Decision Regret Scale; FM, family medicine physicians; GM, general practice physicians; HCP, healthcare professionals; NA, not available; RIS-10, 10-item Regret Intensity Scale; SRH, Self-rated health; US, United States of America*.

Assessment of regret was evaluated by different methods, either qualitative or quantitative. Two out of 14 studies used a qualitative approach (41, 42). This method provides rich data on healthcare professionals' diverse experiences of care-related regret. However, the generalizability of the findings is limited to settings and individuals similar to the study. Furthermore, in those studies, a score for the regret intensity cannot be obtained to assess its intra- or intervariability. This is a critical aspect given the self-reported nature of the data and the potential sample bias (38, 41, 42).

Among quantitative studies, 6 out of 14 studies assessed the intensity of regret by a single question using a Likert scale (11, 12, 36, 38, 43, 44). We also observed a high heterogeneity among the measurements and magnitude of regret across studies (ranging from 1.70 to 8.75 in the RIS-10 or Decision Regret Scales).

A negative correlation was found between regret and the tendency to analytical thinking in a bivariate analysis (36). In a multivariate analysis of the same study, the authors described a positive correlation between regret and maximization, and a negative correlation with satisfaction (36). Interestingly, the authors found no significant relationship between regret and age, despite its strong association with years of experience (9). In contrast, Ben-Ezra and Bibi obtained contrary results, revealing that psychological distress and being younger were associated with higher decision regret (37). Moreover, regret score was dependent on the amount of time elapsed since the regret event asked about had happened (38). Further and more comprehensive studies are needed to better understand the association between care-related regret and demographic and emotional factors.

## Discussion

The DECISIONS-MS is a non-interventional study to assess the impact of care-related regret on healthcare providers managing patients with MS. We propose a standardized approach to investigate care-related regret in the medical field after completing an exhaustive literature review and selecting validated behavioral tools to explore different dimensions of regret. We applied a comprehensive framework to account for different domains relevant to participants' social life.

The dual process theory (DPT) suggests that human decisions are governed by two distinct processes, commonly referred to as system one (intuitive) and system two (analytical) (45). In brief, system one refers to an automatic, unconscious, fast, and effortless (or routine) mechanism to make most common decisions. Conversely, system two makes deliberate decisions, which are non-programmed, conscious, usually slow, and deliberate. Under the DPT framework, it has been suggested that most cognitive biases are attributed to intuitive processes (representing the overuse of system one), or when system one overrides system two (46–48). In this framework, techniques that enhance system two (e.g., a successful educational intervention to overcome regret-related to therapeutic inertia) could counteract these biases, and thereby improve diagnostic accuracy and decrease the likelihood of suboptimal decisions and medical errors (e.g., therapeutic inertia) (49, 50). In this context, care-related regret could be more commonly explained when prompt or automatic decisions (system one) are made by healthcare providers. The development of a perceived suboptimal choice or evidence of a bad outcome (e.g., discovering an error in the disease-modifying treatment administration, more frequent clinical relapses or worsening in the disability, and neuroimaging showing disease progression) may trigger a re-evaluation of the former therapeutic decision that after deliberation (system two) leads to care-related regret.

Regret is associated with suboptimal choices by healthcare professionals (36). It occurs in medical situations of uncertainty, when the probability of an event or outcome is unknown or not 100% certain. Almost 64% of a sample of 494 healthcare professionals in Switzerland reported having experienced a regret in the last 5 years (33). A similar prevalence was identified in a study evaluating American and French physicians (32). Physicians' attitudes toward regret can affect their diagnostic and treatment recommendations (36, 51). Regret was associated with physicians' decisions to order prostate-specific antigen tests and not prescribe anticoagulation in patients with non-valvular atrial fibrillation (36, 52). A survey with a participation of 775 nurses and physicians showed that the intensity of regret in the previous 5 years was associated with poor self-rated health and with higher sick leave among nurses (9). Richner et al. reported that higher regret intensity and more frequent use of maladaptive coping strategies were associated with more sleep difficulties and less work satisfaction (33). Job dissatisfaction and the intention to quit patient care were associated with regrets and maladaptive strategies in a sample of 229 young healthcare professionals (48% nurses) from the Impact of CAre-related Regret Upon Sleep (ICARUS) international study (11).

Regret is an underinvestigated characteristic of healthcare professionals influencing future decisions. Furthermore, higher levels of regret were associated with more difficulties making choices (53, 54). The higher the number of choices or difficulty making decisions faced by physicians, the higher the degree of regret (36). The promotion of evidence-based reflective reasoning or the use of effective interventions in medical education may be useful to ameliorate care-related regret and its impact on diagnostic and treatment decisions (55, 56).

Our protocol has limitations that deserve mention. For example, the RIS-10 has a large time window (5 years) to assess regret. However, a shorter time period may be preferable to overcome recall bias. We also acknowledge the limitations associated with observational studies and the expected variability within and between countries due to cultural, educational, and socio-economic characteristics of participants. Finally, we acknowledge that increased response burden may be associated with lower response rates (57, 58). The study design team took into account this problem increasing the sample size and including a minimally disruptive set of questionnaires without affecting their validity and reliability. Despite these limitations, our study provides a comprehensive strategy to investigate care-related regret among practicing health care professionals. We are looking forward to starting data collection to foster further similar studies in other countries. We hope the results of these studies will inform about socio-economic and behavioral characteristics to ameliorate care-related regret among clinicians and other healthcare professionals.

In conclusion, given that regret is a complex emotion and the literature search shows heterogeneity of methods and conflicting results in assessing care-related regret among healthcare professionals, we propose a comprehensive behavioral battery that includes measures of past and recent regret in different domains and associated behavioral aspects.

## Ethics Statement

The studies involving human participants were reviewed and approved by the ethical board of the Hospital Clínico San Carlos (Madrid, Spain). The participants will provide their written informed consent to participate in this study.

## Author Contributions

GS, GB-G, and JM wrote the study design. APS, AR-A, BR, MB, MT, and JB made significant contributions to the study design. All authors critically revised and approved the manuscript.

## Conflict of Interest

GB-G and JM are employees of Roche Farma Spain. GS reports receiving operating grants from Roche Canada and Spain and Servier Canada and being supported by the Heart and Stroke Foundation of Canada Scientist Award. The remaining authors declare that the research was conducted in the absence of any commercial or financial relationships that could be construed as a potential conflict of interest.
